# Supplementary material for: Chromothripsis during telomere crisis is independent of NHEJ, and consistent with a replicative origin
Source: Genome Res. 2019 May;29(5):737–49. doi: 10.1101/gr.240705.118 (PMC6499312; doi:10.1101/gr.240705.118)
Supplement: Supplemental Material [file supp_gr.240705.118_Supplemental_file_1.zip › contigs/annotated_contigs/DB53/contig.2.DB53_length_275_mean_cov_5.49454545455.docx]

**DB53_length_275_mean_cov_5.49454545455**

```
CCCAGAAAGAGAAAGTAACAAGGAACAAAGAAAACACCACAACAAA|CCAACCTGGGTGACAGAATAAAAAAAAAAAAAAA|A|AAAAA
                                               >chr10:108470173-108470207 + E=4e-03 p=
AAAAAAAAAGCACAGGTCCCAACCAATTTTTTAACTACTTGTATACATATATCAAAATTGTCTAATGTACCACTACACTCCAGCCTGGA
0e+00  >chr8:131399786-131399949 + E=2e-78
TGACAGAGCGAGACTCCATCTCAGAAAAAAAAAAAGCCCAAGTCCCAACCAACTTTTTAACCACT|CTTATATATACAGCAAAATTTTC

TATTTCATTAAA
```
